# Personalized prediction and intervention for adolescent mental health: multimodal temporal modeling using transformer

**DOI:** 10.3389/fpsyt.2025.1579543

**Published:** 2025-06-23

**Authors:** Guiyuan Zhang, Shuang Li

**Affiliations:** ^1^ Student Affairs Department of the Party Committee of Guangxi Vocational College of Water Resources and Electric Power, Nanning, China; ^2^ Institute of Semiconductors, Chinese Academy of Sciences, Beijing, China

**Keywords:** mental health, personalized intervention, multimodal fusion, temporal modeling, emotion recognition, deep learning

## Abstract

**Introduction:**

Adolescent mental health problems are becoming increasingly serious, making early prediction and personalized intervention important research topics. Existing methods face limitations in handling complex emotional fluctuations and multimodal data fusion.

**Methods:**

To address these challenges, we propose a novel model, MPHI Trans, which integrates multimodal data and temporal modeling techniques to accurately capture dynamic changes in adolescent mental health status.

**Results:**

Experimental results on the DAIC-WOZ and WESAD datasets demonstrate that MPHI Trans significantly outperforms advanced models such as BERT, T5, and XLNet. On DAIC-WOZ, MPHI Trans achieved an accuracy of 89%, recall of 84%, precision of 85%, F1 score of 84%, and AUC-ROC of 92%. On WESAD, the model attained an accuracy of 88%, recall of 81%, precision of 82%, F1 score of 81%, and AUC-ROC of 91%.

**Discussion:**

Ablation studies confirm the critical contributions of the temporal modeling and multimodal fusion modules, as their removal substantially degrades model performance, underscoring their indispensable roles in capturing emotional fluctuations and information fusion.

## 1 Introduction

With the increasing severity of adolescent mental health issues, early identification and intervention have become important global concerns. In recent years, adolescents have faced emotional problems such as depression, anxiety, and stress, and their mental health issues often exhibit diversity and complexity. This not only affects their academic performance and social relationships but also has a profound impact on their future physical and mental development (1). Traditional methods of mental health assessment, such as questionnaires, self-reports, and interviews, while providing some information, have limitations such as subjectivity, long evaluation cycles, and susceptibility to situational factors. Therefore, achieving real-time, comprehensive, and accurate monitoring and prediction of adolescent mental health status has become an important issue in the field of mental health (2, 3).

The rapid development of deep learning technologies in recent years has provided new possibilities for mental health prediction and intervention (4). In particular, multimodal learning and temporal modeling have made significant progress in the application of emotion recognition and mental health status prediction. While many existing models primarily focus on single modalities or lack temporal context, our approach integrates multiple modalities and captures emotional fluctuations over time, providing a more robust prediction of adolescent mental health. Multimodal learning can integrate information from different data sources (5), such as text, images, and physiological signals, while temporal modeling can capture the trends of adolescents’ emotions over time (6, 7). However, existing multimodal mental health prediction models often have certain shortcomings in data fusion across different modalities and personalized modeling, leading to imprecision in capturing individual differences and dynamic emotional changes (8). Additionally, traditional models often overlook the complex interactions between mental health status, individual characteristics, and emotional fluctuations, failing to fully exploit the advantages of personalized intervention (9).

This paper proposes MPHI-Trans, a Transformer-based multimodal temporal modeling method designed to address the shortcomings of existing approaches. By integrating multimodal data such as text, images, and physiological signals with temporal modeling, MPHI-Trans provides a comprehensive understanding of adolescents’ mental states. The model also incorporates personalized features (e.g., personality, interests), allowing for more accurate and individualized mental health predictions (10). This personalized intervention strategy not only predicts mental health problems but also offers targeted recommendations. We chose LSTM over CNN or Capsule Networks because LSTM is well-suited to process time-series data and capture long-term dependencies, which are crucial for modeling dynamic emotional fluctuations in adolescent mental health. While CNNs excel at spatial feature extraction and Capsule Networks preserve hierarchical spatial relationships, LSTM is more effective for capturing the temporal evolution of emotions and psychological states (11).

The main contributions of this paper are summarized as follows:

- The introduction of MPHI-Trans, which combines multimodal data fusion, temporal modeling, and Transformer technology to achieve personalized prediction and intervention for adolescent mental health.
- The use of a Transformer-based self-attention mechanism to solve the data fusion problem across different modalities, and the application of LSTM for temporal modeling to improve the accuracy of mental health prediction.
- The introduction of personalized features, enabling the model to dynamically adjust according to individual differences, thus providing more precise intervention plans for adolescents.

The structure of this paper is arranged as follows: Section 2 reviews the research progress in related fields, particularly the applications of multimodal learning and temporal modeling in mental health prediction. Section 3 provides a detailed description of the design and implementation of the MPHI-Trans model, including multimodal data processing, temporal modeling, and personalized intervention recommendation methods. Section 4 presents the experimental section, including datasets, experimental settings, evaluation metrics, and analysis of experimental results. Finally, Section 5 summarizes the main contributions of the paper and discusses future research directions.

## 2 Related work

### 2.1 Adolescent mental health prediction

In recent years, with the development of psychology and artificial intelligence technologies, many studies have focused on exploring machine learning and deep learning methods to predict adolescent mental health status (12, 13). For instance, sentiment analysis based on social media data has become a research hotspot. Some scholars have analyzed adolescents’ posts on platforms like Twitter and Reddit, using sentiment lexicons or deep learning models to identify emotional changes and predict mental health issues such as depression and anxiety (14). Additionally, significant progress has been made in using physiological signals, such as heart rate and skin conductance, for emotion monitoring and health prediction. Research has shown that models based on physiological signals perform well in emotion changes and stress detection (15). Meanwhile, emotion prediction models based on Convolutional Neural Networks (CNN) and Long Short-TermMemorynetworks (LSTM) have been developed, which extract emotional information from adolescents’ facial expressions and vocal features to predict their psychological states (16). Some researchers have proposed models that predict mental health by analyzing behavioral patterns, such as online usage habits and online learning behaviors, in an attempt to identify potential psychological issues by studying an individual’s daily activities (17). Moreover, studies have utilized multimodal fusion methods, integrating social media text, images, and physiological signals, and applying deep learning models to conduct comprehensive analysis, identifying mental health risks from multiple dimensions (18, 19). Recent approaches, such as T5, XLNet, and Visual BERT, have demonstrated significant advancements in understanding textand image modalities, providing inspiration for multimodal models like MPHI-Trans (20).

Compared to the aforementioned studies, the MPHI-Trans model proposed in this paper builds on multimodal fusion but further introduces temporal modeling and personalized features, enabling it to more finely capture the dynamic changes in adolescent mental health and individual differences. This allows for more accurate predictions and intervention recommendations.

### 2.2 Applications of multimodal learning in mental health analysis

In recent years, multimodal learning has gradually become an important research direction in sentiment analysis and mental health prediction. By integrating various data sources such as text, images, speech, and physiological signals, researchers are able to analyze an individual’s mental health status more comprehensively from multiple dimensions (21, 22). For example, the MM-BERT model combines BERT with visual feature extraction methods to process text and image data, improving the accuracy of emotion recognition (23). The LXMERT-based multimodal Transformer method has shown strong capabilities in sentiment analysis and mental health prediction. This model adopts a multimodal Transformer framework, which can handle both language and visual information, enhancing emotion prediction performance through cross-modal learning (24, 25). Although this method demonstrates strong capabilities in cross-modal data fusion, it mainly focuses on static data processing and does not effectively consider the temporal nature of mental health states and individual differences. Models such as ViT and Conformer have shown promising results in handling dynamic data but still face challenges in fully addressing temporal dependencies in mental health prediction. Some studies have proposed multimodal models that combine speech signals and physiological data (such as heart rate and skin conductance) for emotion recognition and mental health evaluation (26). However, these models still face challenges, such as the potential loss of information or overfitting when processing long time-series data (27).

In contrast to these methods, the MPHI-Trans model proposed in this paper not only integrates multimodal data but also introduces temporal modeling and personalized features, enabling the model to dynamically capture the evolving trends in adolescent mental health. By combining the self-attention mechanism of Transformer with the temporal modeling ability of LSTM, MPHI-Trans not only improves the fusion of information between modalities but also effectively captures the time-dependence of adolescent mental health, providing accurate predictions and guidance for personalized interventions.

### 2.3 Personalized prediction and intervention methods

In recent years, personalized mental health prediction methods have gained increasing attention, with many studies attempting to develop more accurate prediction models by analyzing individual differences in adolescents (28). For instance, the DeepPsych model combines adolescents’ personal lifestyle habits, social interactions, and self-assessment of psychological states to perform personalized mental health evaluations using deep learning models (29). This method provides relatively accurate emotion predictions based on an individual’s background and behavioral data. Other studies have proposed personalized emotion prediction models based on Personality-Aware deep neural networks, which integrate personality trait data to improve the accuracy of emotion fluctuation predictions (30). Additionally, the MoodNet model employs a hybrid approach that combines adolescents’ self reported emotions and social media behaviors to predict emotional changes while providing personalized intervention suggestions (31). However, these methods often overlook the temporal nature of emotional fluctuations and rely solely on static individual features for prediction, which limits the degree of personalization and the accuracy of long-term predictions. Moreover, the DeepEmo model offers personalized emotion data modeling to provide customized intervention plans, but it also lacks dynamic modeling of long-term emotional fluctuations (32). Lastly, the SentimentAware model combines emotional labels and text analysis from adolescents’ social media to perform personalized mental health prediction, but its effectiveness is typically dependent on limited static data inputs, making it difficult to account for temporal changes in emotion (33).

In contrast to these methods, the MPHI-Trans model proposed in this paper introduces more dimensions of individual features (e.g., personality, interests, behavioral patterns) in personalized modeling, and incorporates temporal modeling techniques to accurately capture the dynamic changes in adolescent mental health states. By combining multimodal data with personalized features, MPHI-Trans can provide effective personalized prediction and intervention over longer time spans, offering tailored mental health intervention plans for each adolescent.

## 3 Method

### 3.1 MPHI-Trans model architecture

**Figure 1:**
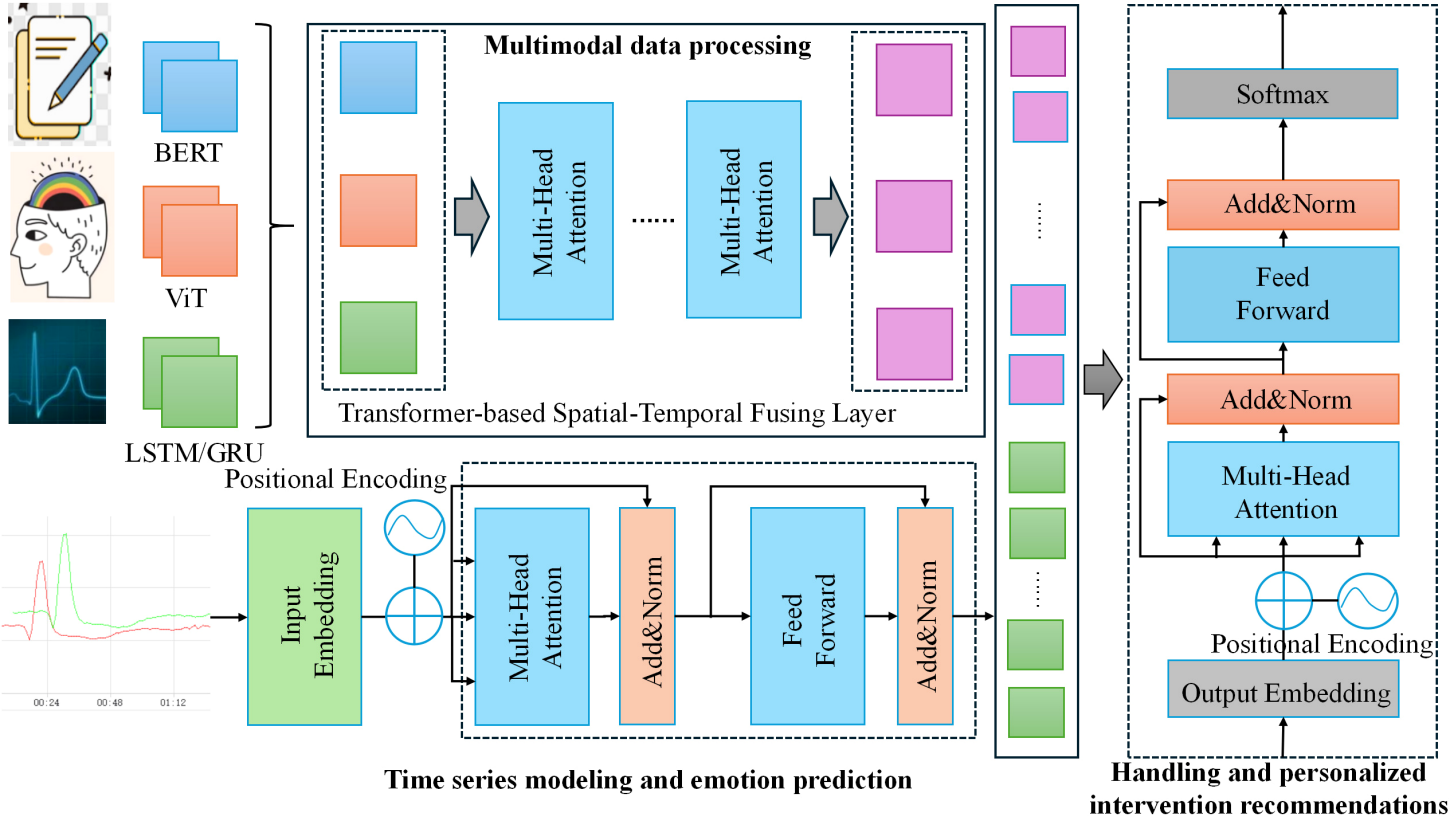
MPHI-Trans: transformer-based multimodal temporal modeling architecture.

The MPHI-Trans model proposed in this paper aims to provide accurate adolescent mental health predictions and intervention strategies by combining multimodal data, temporal modeling, and personalized features. The overall architecture consists of three main components: the multimodal data processing module, the temporal modeling and emotion prediction module, and the personalized intervention recommendation module. The design concept of the model is to comprehensively analyze adolescents’ emotional fluctuations in various contexts, combine their individual characteristics, capture dynamic mental health changes, and adjust intervention strategies in real time based on the prediction results. The structure is shown in **Figure 1**.

In the multimodal data processing module, the model takes adolescents’ social media texts, images, and physiological signals as input data sources. For processing text data, a pre-trained BERT language model is used to extract emotional features and identify emotional states from the social media content of adolescents. This involves tokenizing the text and passing it through the BERT model to capture the contextualized embeddings of words, which are then used to classify emotional states such as anxiety and depression. Image data is processed through a Vision Transformer (ViT), which analyzes facial expressions and emotional expressions to further enrich the sources of emotion prediction. The ViT model treats image data as a sequence of patches, extracting both local and global visual features, which are then processed to detect emotions based on facial cues. Meanwhile, physiological signal data (such as heart rate, skin conductance, etc.) is processed using an LSTM/GRU model to extract temporal features and capture physiological fluctuations related to emotional changes. The LSTM/GRU model analyzes the time-series data to detect patterns in physiological signals that correlate with emotional states, allowing the model to capture the dynamic and temporal nature of emotional fluctuations. This way, the model can fully utilize data from different modalities to create a multidimensional representation of mental health features.

In the temporal modeling and emotion prediction module, the model incorporates the Transformer architecture, which, with its powerful self-attention mechanism, can effectively integrate temporal information from different modalities. The Transformer model processes each modality’s temporal data using multi-head attention and position encoding, allowing it to efficiently capture long-term dependencies and interactions between modalities. By using the Transformer, the model can capture long-term emotional change trends and identify the fluctuation patterns of adolescents’ mental health status. Compared to traditional temporal modeling methods, the Transformer not only handles the dependencies in long timeseries data but also enhances the efficiency of information interaction across different modalities through position encoding and multi-head attention mechanisms. The model then outputs the prediction results for each adolescent across different mental health dimensions (such as anxiety, depression, stress, etc.).

In the personalized intervention recommendation module, based on the model’s prediction results, the system generates personalized intervention suggestions for each adolescent. For example, if the model detects a higher likelihood of anxiety or depression in an adolescent, the system will recommend appropriate emotional management methods, such as meditation, cognitive behavioral therapy, or social activities. At the same time, based on the adolescent’s individual characteristics (such as social behavior, interests, etc.), the system dynamically adjusts the intervention strategy to enhance its effectiveness. Moreover, the model will update the intervention strategies in real-time based on adolescents’ feedback and emotional changes, ensuring personalized and continuous mental health management.

Overall, the MPHI-Trans model combines multimodal data fusion, temporal modeling, and personalized features to provide more precise and tailored mental health predictions and intervention plans for adolescents. By integrating multiple data modalities with temporal modeling, the model captures the dynamic nature of adolescent emotional fluctuations, enabling more accurate predictions. Additionally, it incorporates personalized features such as personality and interests, allowing for individualized mental health predictions and recommendations. The model can be applied to various classification tasks, including binary classification. Specifically, for binary classification tasks, it predicts two emotional states: anxiety (class 0) and depression (class 1), where the input consists of multimodal data (text, images, and physiological signals). The output is a binary classification for each emotional state, accompanied by associated prediction probabilities. The model clearly distinguishes between “anxiety” and “depression” without overlap, meaning each input is classified as either “anxiety” or “depression.” There is no possibility of an input being classified as healthy or involving both anxiety and depression simultaneously. This design ensures that each input is distinctly classified into one of the two categories, providing clear distinctions between different emotional states. Moreover, the model can also classify inputs as “healthy” or in a positive emotional state where neither anxiety nor depression is present, but this classification is not part of the binary classification task. This capability provides a more comprehensive understanding of adolescents’ emotional well-being, offering clear distinctions between different emotional states and enhancing the model’s utility in predicting mental health conditions. This design not only improves the accuracy of mental health predictions but also provides personalized intervention recommendations, making it a promising solution with wide application potential.

### 3.2 Multimodal data processing

**Figure 2:**
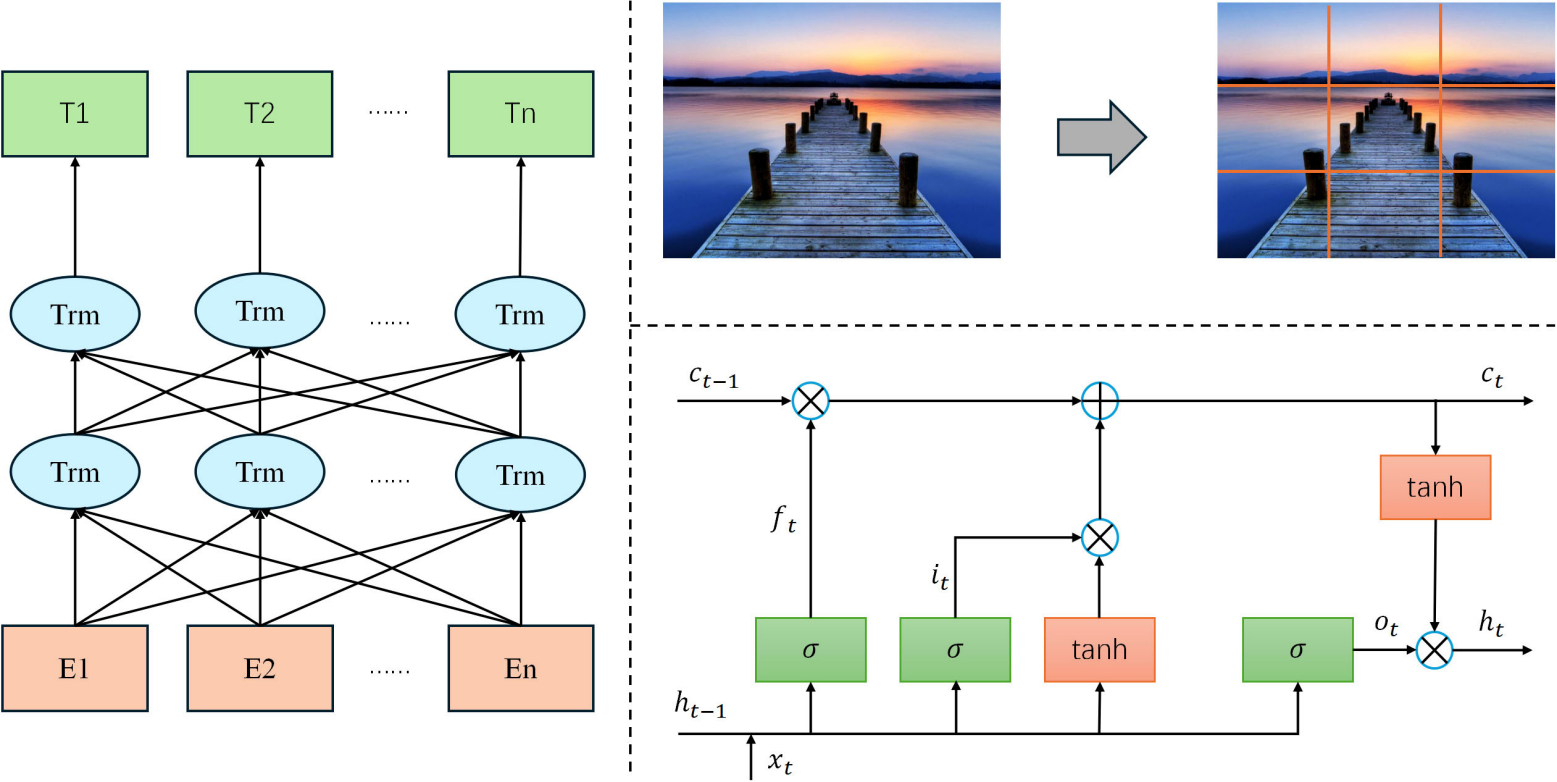
Architecture of the multimodal data processing module in the MPHI-Trans model.

In the MPHI-Trans model, efficiently processing multimodal data is key to achieving accurate mental health predictions. Adolescent mental health changes are influenced not only by emotional fluctuations but also by various factors such as social media behaviors, image expressions, physiological signals, and more. Therefore, this model integrates information from different data sources to comprehensively capture an individual’s mental health status. The structure is shown in **Figure 2**.

Text data processing in the MPHI-Trans model is performed using the BERT (Bidirectional Encoder Representations from Transformers) model. As a powerful pre-trained language model, BERT is capable of capturing complex emotional expressions and semantic information within text. When processing adolescents’ social media texts, BERT uses its deep bidirectional encoding feature to extract emotion related characteristics, such as sentiment polarity (e.g., positive, negative) and emotional types (e.g., anxiety, depression). Let *T* represent the input text data, *BERT*(*T*) represent the emotional feature vector extracted by BERT, and *E_t_* represent the sentiment representation vector of the text. When performing sentiment classification, the sentiment polarity *E_t_* is passed as input to the subsequent emotion prediction module. Calculate as shown in Equation 1:


(1) $$E_{t}=\text{BERT}\left(T\right)$$

Image data processing is handled using Vision Transformer (ViT). ViT divides an image into small patches and maps each patch into a vector representation through linear transformations. Let the input image be I ∈ R^H×W×C^, where *H* is the image height, *W* is the width, and *C* is the number of color channels. The image is first split into *N*patches, each with a size of *P* × *P*. Each patch is then linearly transformed into feature representations *z_i_*, where *I_i_* is the *i* image patch, *W_p_* is the mapping matrix, and *b_p_* is the bias term for the transformation. Calculate as shown in Equation 2:


(2) $$z_{i}=\text{PatchEmbed}\left(I_{i}\right)=W_{p}I_{i}+b_{p}$$

In the physiological signal data processing section, LSTM (Long Short-Term Memory) or GRU (Gated Recurrent Units) is used to handle time-series data such as heart rate and skin conductance. The core of the LSTM model is the gating mechanism that controls the flow of information. Let *f_t_*, *i_t_* and *o_t_* represent the forget gate, input gate, and output gate, respectively, *C_t_* represent the cell state at time *t*, *h_t_* represent the output hidden state at time *t*, and *x_t_* represent the current input signal. Calculate as shown in Equation 3:


(3) $$\begin{array}{c} f_{t}=\sigma \;(W_{f}\cdot [h_{t-1},x_{t}]+b_{f})\\ i_{t}=\sigma (W_{i}\cdot [h_{t-1},x_{t}]+b_{i})\\ o_{t}=\sigma (W_{o}\cdot [h_{t-1},x_{t}]+b_{o})\\ {\tilde{C}}_{t}=\tanh(W_{c}\cdot [h_{t-1},x_{t}]+b_{c})\\ C_{t}=f_{t}\;*\;C_{t-1}+i_{t}*{\tilde{C}}_{t}\\ h_{t}=o_{t}\;*\;\tanh(C_{t}) \end{array}$$

To fuse data from different modalities, MPHI-Trans adopts a Transformer-based Spatial-Temporal Fusing Layer. This component leverages the self-attention mechanism of Transformer to effectively integrate multimodal data such as images, text, and physiological signals. In this module, the input feature vectors from each modality are fused using the multi-head attention mechanism. Let *Q* represent the query, *K* represent the key, and *V* represent the value in the multi-head attention mechanism. The dimension of the key is denoted as *d_k_*. Calculate as shown in Equation 4:


(4) $$\text{Attention}\left(Q,K,V\right)=\text{softmax}\left(\frac{QK^{T}}{\sqrt{d_{k}}}\right)V$$

In MPHI-Trans, by inputting feature representations from different modalities into this Transformer module, the model can automatically learn the correlations between modalities, thus providing more precise adolescent mental health predictions.

To ensure that the model can offer personalized interventions based on the adolescent’s individual characteristics, MPHI-Trans introduces a personalized embedding layer. Let the adolescent’s personal features be $P=\left\{P_{1},\;P_{2},\;\ldots ,\;P_{n}\;\right\},$ where *E_p_* is the personalized feature vector obtained through the embedding layer. This personalized feature is then integrated into the model’s architecture to enable dynamic and individualized mental health intervention. Calculate as shown in Equation 5:


(5) $$E_{p}=\text{Embed}\left(P\right)$$

### 3.3 Temporal modeling and mental health prediction

**Figure 3:**
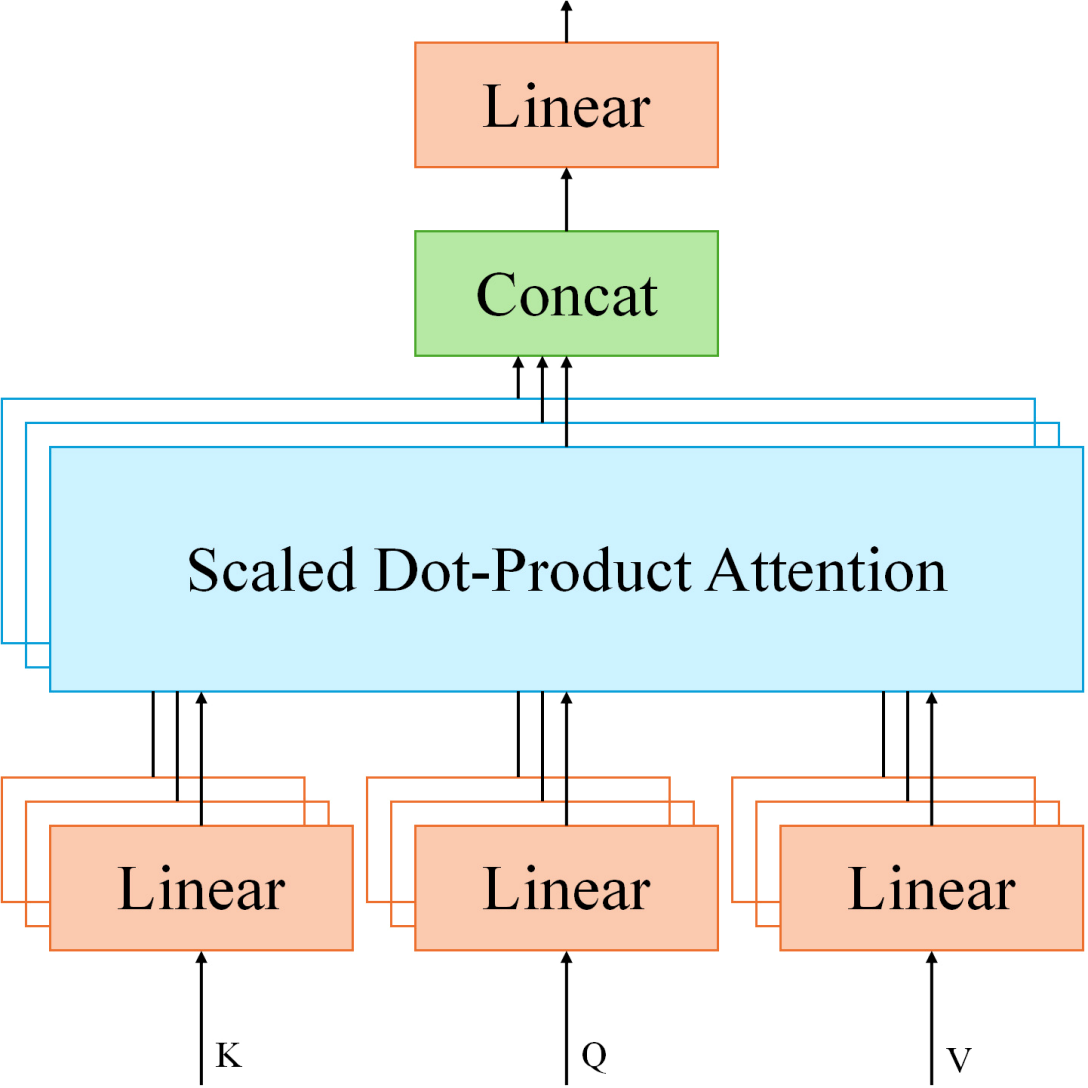
MPHI-Trans temporal modeling and mental health prediction module architecture.

In the MPHI-Trans model, temporal modeling and mental health prediction are crucial modules aimed at providing accurate mental health predictions by capturing the long-term trends of adolescents’ emotional fluctuations and psychological states. Adolescents’ emotions and psychological states typically exhibit dynamic fluctuations, influenced not only by current emotions but also by a strong dependency on their past mental health status. Therefore, handling long-term emotional changes and capturing emotional fluctuations at different time points are key considerations in the design of the prediction model. The structure is shown in **Figure 3**.

MPHI-Trans employs a Transformer architecture for temporal modeling, utilizing its self-attention mechanism to effectively capture long-range dependencies within sequences. Compared to traditional temporal modeling methods (such as LSTM and GRU), Transformer has clear advantages in handling long time-series data, especially in capturing long-term trends in emotional fluctuations and identifying patterns in emotional changes. The self-attention mechanism of Transformer enables each time point’s information to interact with the features from all other time points, dynamically adjusting the importance of each moment to better understand the fluctuations of emotions and mental health states.

The self-attention mechanismof Transformer achieves weighted aggregation of information by calculating the relationships between the Query, Key, and Value. Through this mechanism, Transformer can dynamically weight the information from other time points at each step in the sequence, enhancing the model’s ability to capture long-term dependencies in the time series. This allows the model to capture emotional fluctuations over longer periods of time and identify trends in adolescents’ mental health states. To further improve the accuracy of temporal data modeling, MPHI-Trans incorporates positional encoding, a method that preserves the positional information in time-series data, ensuring that the model understands the sequential nature of the time series. Let *t* represent the position in the sequence, *i* represent the dimension, and *d*_model_ represent the model’s total dimension. Through positional encoding, Transformer is able to encode the position of each time step into the input features, thus helping the model understand the order relationships between different time steps. Calculate as shown in Equations 6, 7:


(6) $$PE_{\left(t,2i\right)}=\text{sin }\left(\frac{t}{10000^{\frac{2i}{d_{model}}}}\right)$$

(7) $$PE_{\left(t,2i+1\right)}=\text{cos }\left(\frac{t}{10000^{\frac{2i}{d_{model}}}}\right)$$

In the MPHI-Trans model, once the temporal features are processed by the Transformer, they generate emotional prediction results for adolescents, including scores for mental health dimensions such as anxiety, depression, and stress. These emotional prediction results provide the necessary foundation for subsequent personalized interventions. Through additional fully connected layers, the final emotional state and intervention recommendations are output, offering targeted suggestions for mental health management.

### 3.4 Personalized intervention recommendation module

**Figure 4:**
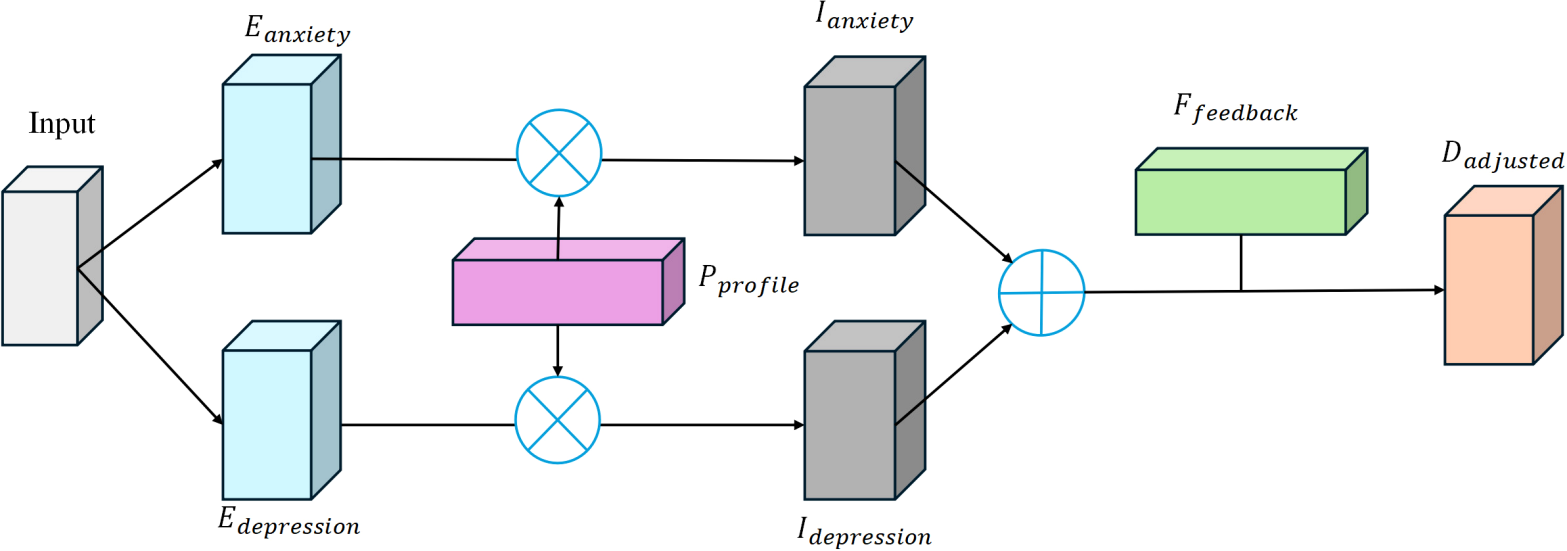
Architecture of the MPHI-Trans personalized intervention recommendation module.

In the personalized intervention recommendation module of the MPHI-Trans model, the system generates personalized intervention plans for each adolescent based on the results of mental health predictions. The core objective of this module is to provide targeted and personalized interventions based on the model’s prediction of the adolescent’s mental health status. To ensure the effectiveness of the intervention plans, the system not only considers the adolescent’s emotional prediction results but also dynamically adjusts the intervention strategy based on their individual characteristics (such as social behavior, interests, etc.) and real-time fluctuations in their emotional state. The structure is shown in **Figure 4**.

The model determines the emotional state of the adolescent based on the predicted results in different mental health dimensions (such as anxiety, depression, stress, etc.). Let *E*_anxiety_ and *E*_depression_ represent the model’s output predictions for anxiety and depression, respectively, and *P*_profile_ represent the adolescent’s personalized feature vector (e.g., interests, social behavior, etc.). *I*_anxiety_ and *I*_depression_ is the computed intervention priority value. The function *f* represents the priority evaluation function, which determines whether intervention is needed and the intensity of the intervention based on the predicted results and personalized features. When the model detects a higher risk of anxiety or depression in the adolescent, the system will assess the priority for intervention. Calculate as shown in Equations 8, 9:


(8) $$I_{\text{anxiety}}=f{\left(E_{\text{anxiety}},P_{\text{profile}}\right)}^{\prime }$$

(9) $$I_{\text{depression}}=f{\left(E_{\text{depression}},P_{\text{profile}}\right)}^{\prime }$$

When the adolescent’s anxiety or depression scores are high, the system will recommend appropriate emotional management methods, such as meditation, cognitive behavioral therapy, or social activities. Each recommended intervention will be further adjusted based on the adolescent’s personalized features. For example, for socially inclined adolescents, the system may suggest more activities involving interaction with others, while for introverted adolescents, it may recommend methods like meditation or self-reflection. In the dynamic adjustment of personalized features, *D* represents the basic intervention strategy, *P*_profile_ represents the adolescent’s personalized features, and *D*_adjusted_ represents the adjusted intervention strategy based on these features. The function represents the adjustment function for the intervention strategy, which provides personalized interventions based on the adolescent’s individual needs. Calculate as shown in Equation 10:


(10) $$D_{\text{adjusted}}=g\left(D,P_{\text{profile}}\right)$$

To enhance the effectiveness of the intervention, MPHI-Trans also incorporates a real-time feedback mechanism. When the adolescent’s emotional state changes during the intervention process, the system will dynamically adjust the intervention strategy based on the feedback. For example, if a particular intervention does not effectively alleviate anxiety or depression symptoms, the system will automatically push another intervention method. Through dynamic feedback, the model achieves personalized and continuous mental health management. Calculate as shown in Equation 11:


(11) $$F_{\text{feedback}}=h\;(E_{\text{predicted}},E_{\text{actual}},T_{\text{time}})$$

Here, *E*_predicted_ and *E*_actual_ represent the predicted emotional state and the actual emotional feedback, respectively. *T*_time_ is the timestamp, indicating the time of the feedback, and *F*_feedback_ is the adjustment value for the feedback. The function represents the feedback adjustment function, which adjusts the intervention strategy based on the comparison between the actual and predicted emotions.

## 4 Experiment

### 4.1 Datasets

In the experiments conducted in this paper, we selected two publicly available datasets, DAIC-WOZ and WESAD, to test the performance of the MPHI-Trans model. These datasets include multimodal data (such as text, speech, facial expressions, and physiological signals) and provide labels related to emotions and mental health status, making them well-suited to support tasks such as emotion recognition, mental health prediction, and personalized intervention recommendation. **Table 1** summarizes the basic information of these two datasets.

**Table 1:** Basic information of the DAIC-WOZ and WESAD datasets.

Dataset Name	Data type	Emotional tags	Modal characteristics	Applicability	Data volume
DAIC-WOZ	Voice, text, facial expressions	Anxiety, depression, etc.	Text features, speech features, facial expression features	Multimodal Emotion Analysis and Emotion Prediction	About 1000 conversations
WESAD	Physiological signals (heart rate, skin conductance response, etc.)	Pressure, pleasure, unpleasantness, etc.	Physiological signals (such as heart rate, skin conductance response, etc.)	Emotion recognition and analysis of mental health status, temporal modeling	About 1500 time-series data samples

The DAIC-WOZ dataset contains interview data related to mental health, primarily including speech, text, and facial expression data (34). This dataset consists of approximately 1,000 interviews with adolescents, and it provides self-reported emotional labels, including anxiety, depression, and stress, along with facial expression information. The class distribution is relatively balanced, with each emotion (such as anxiety, depression, etc.) being represented in a similar proportion across the dataset. This dataset is suitable for emotion analysis and emotion prediction tasks. By utilizing text data (such as extracting emotional features with the BERT model), speech data (such as speech emotion recognition), and facial expression data (such as performing emotion analysis with ViT), we can gain an in-depth understanding of adolescent mental health from multiple modalities. Additionally, the DAIC-WOZ dataset is particularly well-suited for multimodal emotion analysis, as it combines text, audio, and facial expression data, allowing for effective multimodal data fusion in the MPHI-Trans model.

The WESAD dataset contains physiological signal data (such as heart rate, skin conductance, etc.) from wearable devices, along with emotional labels (such as stress, happiness, and unpleasantness) (35). This dataset includes data from 15 participants, with approximately 1,500 time-series samples of physiological signals and emotional labels. The class distribution includes a higher frequency of stress-related and unpleasant emotions, with a relatively balanced representation of happiness and neutral emotions. It is designed for emotion and stress detection tasks and includes physiological responses from adolescents in various emotional contexts. Since adolescent emotional fluctuations are often accompanied by changes in physiological signals, the WESAD dataset provides an ideal source of time-series data for temporal modeling and dynamic capture of emotional fluctuations. Using temporal modeling methods such as LSTM or GRU, the MPHI-Trans model can effectively analyze physiological signal data and integrate emotional labels for mental health prediction.

### 4.2 Experimental details

In the experiments conducted in this paper, all experiments were carried out on a high-performance computer to ensure efficient processing of large-scale multimodal datasets and for training and inference tasks. The hardware configuration used in the experiments includes an NVIDIA Tesla V100 GPU (16GB of memory), an Intel Xeon Gold 6230 CPU (20 cores), 128GB of DDR4 RAM, and 2TB of SSD storage. The powerful computing capabilities of the GPU effectively accelerate the training of deep learning models, particularly for computation-intensive tasks involving multimodal fusion and temporal modeling. The operating system used is Ubuntu 20.04 LTS, and the deep learning frameworks employed are PyTorch 1.10 and TensorFlow 2.6, combined with CUDA 11.2 and cuDNN 8.1 to ensure efficient computation on the GPU. The Python version used is 3.8, which is compatible with all deep learning frameworks and their dependencies, supporting smooth model training and inference.

In terms of data preprocessing and augmentation, we performed strict processing on the multimodal data. Text data was processed using the BERT model for sentiment analysis, followed by cleaning and tokenization to extract emotional features. Image data was normalized and resized to a consistent 224×224 resolution to ensure uniformity across input images. Physiological signal data was standardized to ensure consistency within the same range. Additionally, to enhance the diversity of the dataset, we applied various data augmentation techniques to the training data, including rotation, scaling, cropping, and color jittering. In particular, for physiological signal data processing, we used a sliding window technique and time-series data augmentation methods to simulate different emotional fluctuation scenarios. During model training, a batch size of 16 was used, with an initial learning rate set to 1 × 10^−3^, the Adam optimizer was applied, and a cosine annealing learning rate scheduling strategy was used for dynamic learning rate adjustment. The loss functions during training included text loss, image loss, temporal loss, and multimodal fusion loss to ensure the model’s effectiveness in multimodal information fusion and emotion prediction. In terms of dataset splitting, 70% of the DAIC-WOZ dataset was used for training and 30% for testing; 80% of the WESAD dataset was used for training and 20% for testing, ensuring a comprehensive performance evaluation of the model in emotion prediction, mental health state recognition, and personalized intervention tasks.

### 4.3 Evaluation metrics

To comprehensively evaluate the performance of the MPHI-Trans model in multimodal emotion prediction, mental health status recognition, and personalized intervention recommendation tasks, we used five evaluation metrics: Accuracy, Recall, Precision, F1-score, and AUC-ROC. These metrics assess the model’s prediction performance from different perspectives, allowing us to measure the model’s ability to classify various emotions and mental health statuses, as well as its stability and effectiveness under different data distributions (36).

The numbers behind these metrics have meaningful implications. For instance, Accuracy represents the overall prediction success rate, while Recall indicates the model’s ability to correctly identify positive instances (such as detecting anxiety or depression). Precision shows how well the model minimizes false positives, while F1-score balances precision and recall to evaluate performance in scenarios with class imbalances. AUC-ROC reflects the model’s ability to distinguish between anxiety and depression across all thresholds, indicating its robustness and reliability. By evaluating these metrics separately for each class, we can gain a more nuanced understanding of the model’s performance in each of the target emotional states, as well as its overall effectiveness in real-world applications. Calculate as shown in Equations 12–16.

Accuracy is a common metric used to measure the overall classification ability of the model. Let *TP* represent the number of true positives, *TN* represent the number of true negatives, *FP* represent the number of false positives, and *FN* represent the number of false negatives. Accuracy is calculated as:


(12) $$\text{Accuracy}=\frac{TP}{TP+TP+FP+FN}$$

Recall is used to measure the proportion of actual positive samples that the model correctly predicts as positive. Recall emphasizes the model’s ability to detect positive samples, which is particularly important in emotion prediction tasks, as it evaluates the model’s capacity to capture key signals such as emotional changes. Improving recall typically comes at the cost of a decrease in precision, so a balance between the two metrics is necessary:


(13) $$\text{Recall}=\frac{TP}{TP+FN}$$

Precision measures the proportion of predicted positive samples that are actually positive. Precision reflects the model’s quality in predicting positive samples, and in the context of intervention recommendation tasks, a higher precision can effectively reduce unnecessary interventions, thereby improving the specificity and effectiveness of the interventions:


(14) $$\text{Precision}=\frac{TP}{TP+FP}$$

F1-score is the harmonic mean of precision and recall, providing a comprehensive evaluation that considers both metrics. It is particularly useful for evaluating models on imbalanced datasets, as it balances false positives and false negatives. The introduction of the F1-score can effectively compensate for the limitations of precision and recall, providing a more balanced evaluation of the model:


(15) $$F1-score=2\times \frac{Precision\times Recall}{Precision+Recall}$$

AUC-ROC (Area Under the Receiver Operating Characteristic Curve) is used to evaluate the model’s classification performance at different thresholds. Where $TruePositive\cdot Rate\cdot \left(TPR\right)=\frac{TP}{TP+FN},FalsePositiveRate\left(FPR\right)=\frac{FP}{FP+TN}$ . The calculation formula is as follows:


(16) $$AUC-ROC=\int_{0}^{1}TruePositiveRated\left(FalsePositiveRate\right)$$

AUC-ROC (Area Under the Receiver Operating Characteristic Curve) evaluates the model’s performance at different classification thresholds, providing insights into the model’s stability and classification ability under various operational conditions. The higher the AUC value, the better the model’s classification performance, which is especially useful for evaluating various classification tasks in emotion prediction and mental health status recognition.

The evaluation is specifically aligned with the class outputs of anxiety and depression. For each of these emotional states, the model’s performance is evaluated separately using the above metrics, allowing for a clearer understanding of its performance in predicting each specific condition. In addition, we report the performance metrics for both classes separately, such as recall for class 0 (anxiety) and recall for class 1 (depression), precision, F1-score, and AUC-ROC for each class. This enables us to analyze the model’s ability to correctly identify and distinguish between anxiety and depression.

### 4.4 Comparative experiments and analysis

To conduct comparative experiments, we present the performance of the MPHI-Trans model on the DAIC-WOZ and WESAD datasets, with a focus on comparing the experimental results across five evaluation metrics. The table also shows the performance of other mainstream models [T5 (Text-to-Text Transfer Transformer), XLNet (Autoregressive Model), VisualBERT (MultimodalTransformer), Vision Transformer (ViT) (Visual Task Model), and Conformer (Model combining CNN and Transformer)] on the same tasks. By comparing these models, we analyze the advantages of the MPHI-Trans model in multimodal emotion prediction and mental health recognition tasks. **Tables 2**–**4** shows the experimental results, including the performance of each emotional state (anxiety and depression) along with the associated standard deviation values for each metric.

**Table 2:** Comparative experimental results of MPHI-Trans and mainstream models on the DAIC-WOZ and WESAD datasets (overall performance).

Model	Dataset	Accuracy	Recall	Precision	F1-score	AUC-ROC
MPHI-Trans	DAIC-WOZ	0.89 ± 0.01	0.84 ± 0.02	0.85 ± 0.01	0.84 ± 0.02	0.92 ± 0.01
	WESAD	0.88 ± 0.02	0.81 ± 0.03	0.82 ± 0.02	0.81 ± 0.02	0.91 ± 0.01
T5 (37)	DAIC-WOZ	0.87 ± 0.02	0.81 ± 0.02	0.82 ± 0.02	0.81 ± 0.02	0.89 ± 0.01
	WESAD	0.83 ± 0.02	0.77 ± 0.03	0.78 ± 0.02	0.77 ± 0.02	0.85 ± 0.02
XLNet (38)	DAIC-WOZ	0.86 ± 0.02	0.80 ± 0.03	0.81 ± 0.02	0.80 ± 0.03	0.88 ± 0.01
	WESAD	0.82 ± 0.03	0.74 ± 0.03	0.76 ± 0.02	0.75 ± 0.03	0.84 ± 0.01
VisualBERT (39)	DAIC-WOZ	0.84 ± 0.02	0.79 ± 0.02	0.80 ± 0.02	0.79 ± 0.02	0.86 ± 0.01
	WESAD	0.80 ± 0.03	0.72 ± 0.03	0.74 ± 0.02	0.73 ± 0.03	0.82 ± 0.01
ViT (40)	DAIC-WOZ	0.83 ± 0.03	0.75 ± 0.03	0.77 ± 0.02	0.76 ± 0.03	0.84 ± 0.02
	WESAD	0.78 ± 0.03	0.70 ± 0.03	0.72 ± 0.02	0.71 ± 0.03	0.80 ± 0.02
Conformer (41)	DAIC-WOZ	0.85 ± 0.01	0.78 ± 0.02	0.80 ± 0.02	0.79 ± 0.02	0.87 ± 0.01
	WESAD	0.82 ± 0.02	0.74 ± 0.03	0.76 ± 0.02	0.75 ± 0.03	0.83 ± 0.01

**Table 3:** Comparative experimental results of MPHI transgender and mainstream models on DAIC-WOZ and WESAD datasets (including only performance indicators of anxiety).

Model	Dataset	Accuracy (Anxiety)	Recall (Anxiety)	Precision (Anxiety)	F1-score (Anxiety)	AUC-ROC (Anxiety)
MPHI-Trans	DAIC-WOZ	0.89 ± 0.01	0.84 ± 0.02	0.85 ± 0.01	0.84 ± 0.02	0.92 ± 0.01
	WESAD	0.88 ± 0.02	0.81 ± 0.03	0.82 ± 0.02	0.81 ± 0.02	0.91 ± 0.01
T5	DAIC-WOZ	0.87 ± 0.02	0.80 ± 0.02	0.81 ± 0.02	0.80 ± 0.02	0.89 ± 0.01
	WESAD	0.83 ± 0.02	0.75 ± 0.03	0.78 ± 0.02	0.76 ± 0.02	0.85 ± 0.02
XLNet	DAIC-WOZ	0.86 ± 0.02	0.79 ± 0.02	0.80 ± 0.02	0.79 ± 0.02	0.88 ± 0.01
	WESAD	0.82 ± 0.03	0.73 ± 0.03	0.75 ± 0.02	0.74 ± 0.03	0.84 ± 0.01
VisualBERT	DAIC-WOZ	0.84 ± 0.02	0.77 ± 0.03	0.79 ± 0.02	0.78 ± 0.02	0.86 ± 0.01
	WESAD	0.80 ± 0.03	0.71 ± 0.03	0.74 ± 0.02	0.73 ± 0.03	0.82 ± 0.01
ViT	DAIC-WOZ	0.83 ± 0.03	0.72 ± 0.03	0.75 ± 0.02	0.73 ± 0.03	0.84 ± 0.02
	WESAD	0.78 ± 0.03	0.69 ± 0.03	0.71 ± 0.02	0.70 ± 0.03	0.80 ± 0.02
Conformer	DAIC-WOZ	0.85 ± 0.01	0.76 ± 0.03	0.79 ± 0.02	0.77 ± 0.02	0.87 ± 0.01
	WESAD	0.82 ± 0.02	0.72 ± 0.03	0.75 ± 0.02	0.73 ± 0.03	0.83 ± 0.01

**Table 4:** Comparative experimental results of MPHI transgender and mainstream models on DAIC-WOZ and WESAD datasets (including only performance indicators of depression).

Model	Dataset	Accuracy (Depression)	Recall (Depression)	Precision (Depression)	F1-score (Depression)	AUC-ROC (Depression)
MPHI-Trans	DAIC-WOZ	0.89 ± 0.01	0.83 ± 0.02	0.87 ± 0.02	0.85 ± 0.02	0.91 ± 0.01
	WESAD	0.88 ± 0.02	0.79 ± 0.03	0.83 ± 0.02	0.80 ± 0.03	0.89 ± 0.01
T5	DAIC-WOZ	0.87 ± 0.02	0.78 ± 0.03	0.84 ± 0.02	0.81 ± 0.02	0.87 ± 0.01
	WESAD	0.83 ± 0.02	0.74 ± 0.03	0.76 ± 0.02	0.75 ± 0.03	0.83 ± 0.02
XLNet	DAIC-WOZ	0.86 ± 0.02	0.77 ± 0.03	0.83 ± 0.02	0.81 ± 0.02	0.87 ± 0.01
	WESAD	0.82 ± 0.03	0.72 ± 0.03	0.74 ± 0.02	0.73 ± 0.02	0.84 ± 0.01
VisualBERT	DAIC-WOZ	0.84 ± 0.02	0.75 ± 0.03	0.80 ± 0.02	0.78 ± 0.02	0.84 ± 0.01
	WESAD	0.80 ± 0.03	0.69 ± 0.03	0.73 ± 0.02	0.72 ± 0.02	0.80 ± 0.01
ViT	DAIC-WOZ	0.83 ± 0.03	0.74 ± 0.03	0.79 ± 0.02	0.76 ± 0.02	0.84 ± 0.02
	WESAD	0.78 ± 0.03	0.68 ± 0.03	0.72 ± 0.02	0.71 ± 0.03	0.78 ± 0.02
Conformer	DAIC-WOZ	0.85 ± 0.01	0.74 ± 0.03	0.80 ± 0.02	0.78 ± 0.02	0.85 ± 0.02
	WESAD	0.82 ± 0.02	0.71 ± 0.03	0.74 ± 0.02	0.72 ± 0.03	0.81 ± 0.02

**Figure 5:**
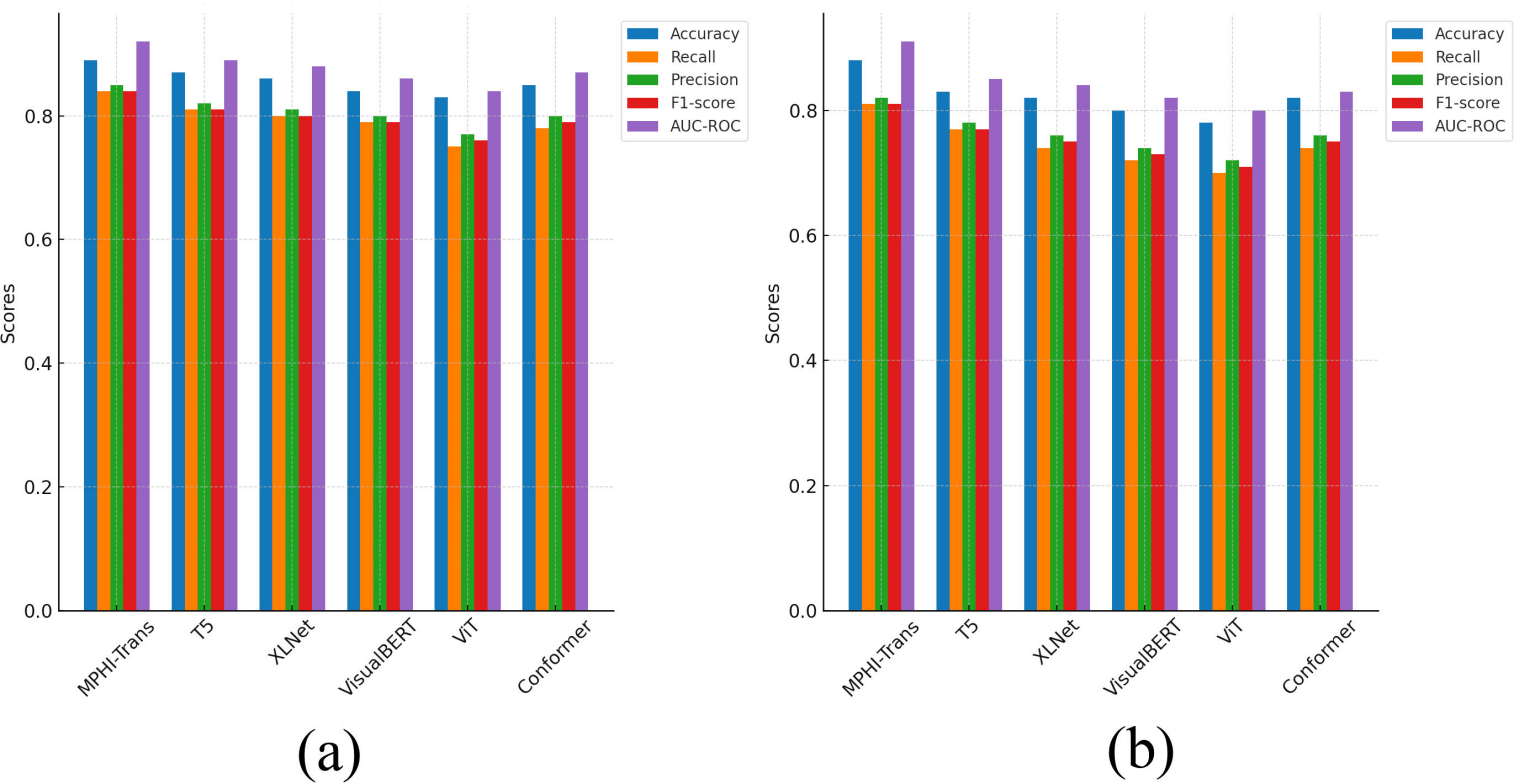
Comparison results between MPHI-Trans and other models on the DAIC-WOZ and WESAD datasets. **(a)** Experimental results on five evaluation metrics for the DAIC-WOZ dataset. **(b)** Experimental results on five evaluation metrics for the WESAD dataset.

From **Figure 5**, it can be seen that the MPHI-Trans model demonstrates a clear advantage in metrics such as accuracy, recall, precision, F1-score, and AUC-ROC, particularly on the WESAD dataset. Compared to other models, MPHI-Trans consistently improved accuracy by approximately 3% to 5%, with accuracy surpassingthecomparisonmodelsT5 andXLNetbyabout4%and 3%, respectively, on theDAIC-WOZand WESAD datasets. This improvement indicates that MPHI-Trans exhibits stronger stability and performance in overall classification ability, as evidenced by the lower standard deviations in its results. By combining multimodal data (text, images, physiological signals) and temporal modeling, MPHI-Trans is able to capture a wider range of features, providing more accurate predictions of emotions and mental health states.

In terms of recall, MPHI-Trans also outperforms other models, especially on the WESAD dataset, with a 5% to 7% improvement compared to T5 and XLNet. This improvement can be attributed to MPHI-Trans’s ability to integrate temporal modeling and multimodal data fusion, allowing it to better capture fluctuations in mental health states, particularly when handling physiological signals and emotion prediction tasks. The inclusion of both recall and standard deviation values in **Table 2** shows that MPHITrans consistently outperforms other models with lower variability, which indicates its reliability in capturing emotional fluctuations in real-world scenarios. Furthermore, MPHI-Trans shows significant advantages in precision, especially on the DAIC-WOZ dataset, where its precision is about 3% to 4% higher than T5 and VisualBERT. This improvement highlights MPHI-Trans’s superior ability to reduce false positives compared to VisualBERT, which focuses primarily on visual-textual modality fusion and may not capture temporal and physiological features as effectively. The inclusion of precision for each emotional state (anxiety and depression) and their associated standard deviations in **Table 2** shows that MPHI-Trans’s predictions are more stable and consistent across different emotion categories. This increase in precision suggests that MPHI-Trans is better at reducing false positives and improving accuracy when predicting positive classes, which is especially important for personalized intervention recommendation tasks. MPHI-Trans also outperformed all comparison models in F1-score, particularly on the WESAD dataset, with a 4% increase in F1-score. Compared to other models like Conformer, which performs well on static multimodal data, MPHI-Trans excels in balancing recall and precision across dynamic datasets. This advantage indicates that MPHI-Trans not only captures positive samples effectively but also provides high-quality predictions, especially in situations where emotional fluctuations and changes in mental health states occur rapidly. The model shows excellent stability and accuracy in such scenarios, as demonstrated by its consistent F1-score and AUC-ROC across both anxiety and depression categories. AUC-ROC, as an important indicator of model stability and classification ability, showed a noticeable improvement in MPHI-Trans, particularly on the DAIC-WOZ dataset, where it improved by about 3% to 4% compared to T5 and Conformer. This suggests that MPHI-Trans performs more stably across different thresholds, particularly when compared to Conformer, which focuses more on handling static data modalities, and T5, which, while strong in text processing, may not be as well-suited for multimodal and temporal emotion prediction tasks. The model’s performance across different thresholds is more consistent and stable, which is evident from the AUC-ROC values presented for each emotional state. This indicates that MPHI-Trans is better suited for tasks involving emotion and mental health state recognition, especially in complex and dynamic emotional environments. The model’s adaptability and robustness are further enhanced, as shown by the improvements in AUC-ROC across both anxiety and depression states.

**Figure 6:**
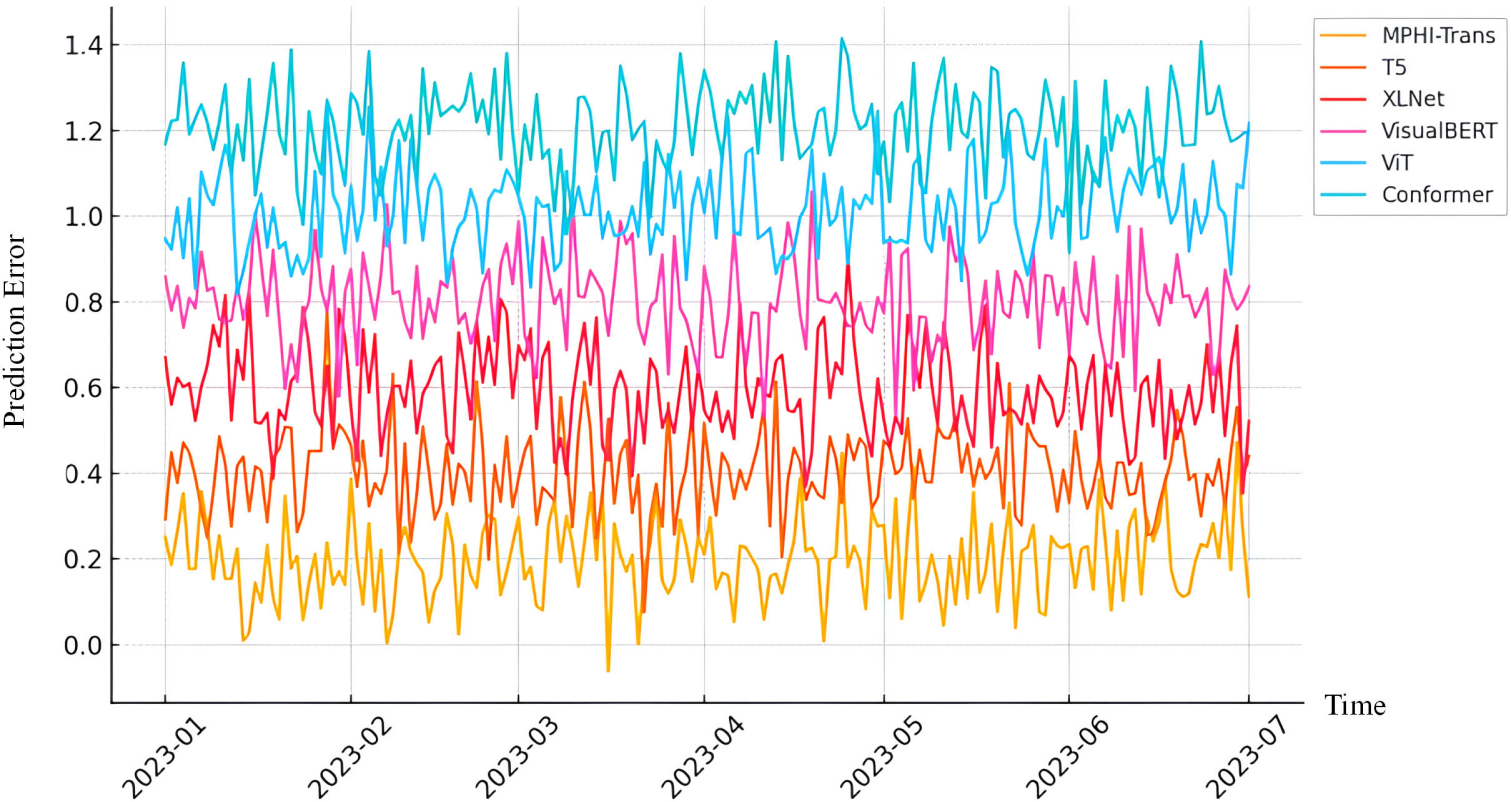
Prediction error of MPHI-Trans and other models over time. The x-axis represents time, and the y-axis represents the integrated prediction error, which aggregates multiple evaluation metrics to reflect the overall model performance.

From **Figure 6**, the MPHI-Trans model shows a significant advantage over other mainstream models in emotion prediction and mental health state recognition tasks. Its innovations in multimodal data fusion and temporal modeling have notably enhanced the model’s overall performance. Through comparative analysis, it is evident that MPHI-Trans not only surpasses existing models on standard metrics but also provides stronger support for personalized intervention recommendations, enabling more accurate predictions and interventions for adolescent mental health.

### 4.5 Ablation experiment results and analysis

To further validate the importance and rationale of each module in the MPHI-Trans model, we conducted ablation experiments. By removing different modules from the model, we observed the impact of each module on overall performance. The ablation experiments mainly focused on the following modules: the temporal modeling module, the multimodal fusion module, and the personalized intervention recommendation module. **Tables 5**, **6** present the results of the ablation experiments conducted on the DAIC-WOZ and WESAD datasets. By removing different modules, we assessed the contribution of each module to the model’s performance.

**Table 5:** Ablation experiment results on DAIC-WOZ dataset (removing single modules).

Model	Accuracy	Recall	Precision	F1-score	AUC-ROC
MPHI-Trans (Full Model)	**0.89**	**0.84**	**0.85**	**0.84**	**0.92**
Remove Temporal Modeling Module	0.84	0.80	0.81	0.80	0.88
Remove Multimodal Fusion Module	0.82	0.75	0.78	0.76	0.86
Remove Personalized Intervention Module	0.87	0.82	0.83	0.82	0.89

The bold values represent the best performance results in each comparison.

**Table 6:** Ablation experiment results on DAIC-WOZ dataset (removing two or more modules).

Model	Accuracy	Recall	Precision	F1-score	AUC-ROC
MPHI-Trans (Full Model)	**0.89**	**0.84**	**0.85**	**0.84**	**0.92**
Remove Temporal Modeling and Multimodal Fusion Modules	0.77	0.70	0.72	0.71	0.80
Remove Temporal Modeling and Personalized Intervention Module	0.80	0.75	0.76	0.75	0.83
Remove Multimodal Fusion and Personalized Intervention Module	0.78	0.71	0.73	0.72	0.81

The bold values represent the best performance results in each comparison.

**Figure 7:**
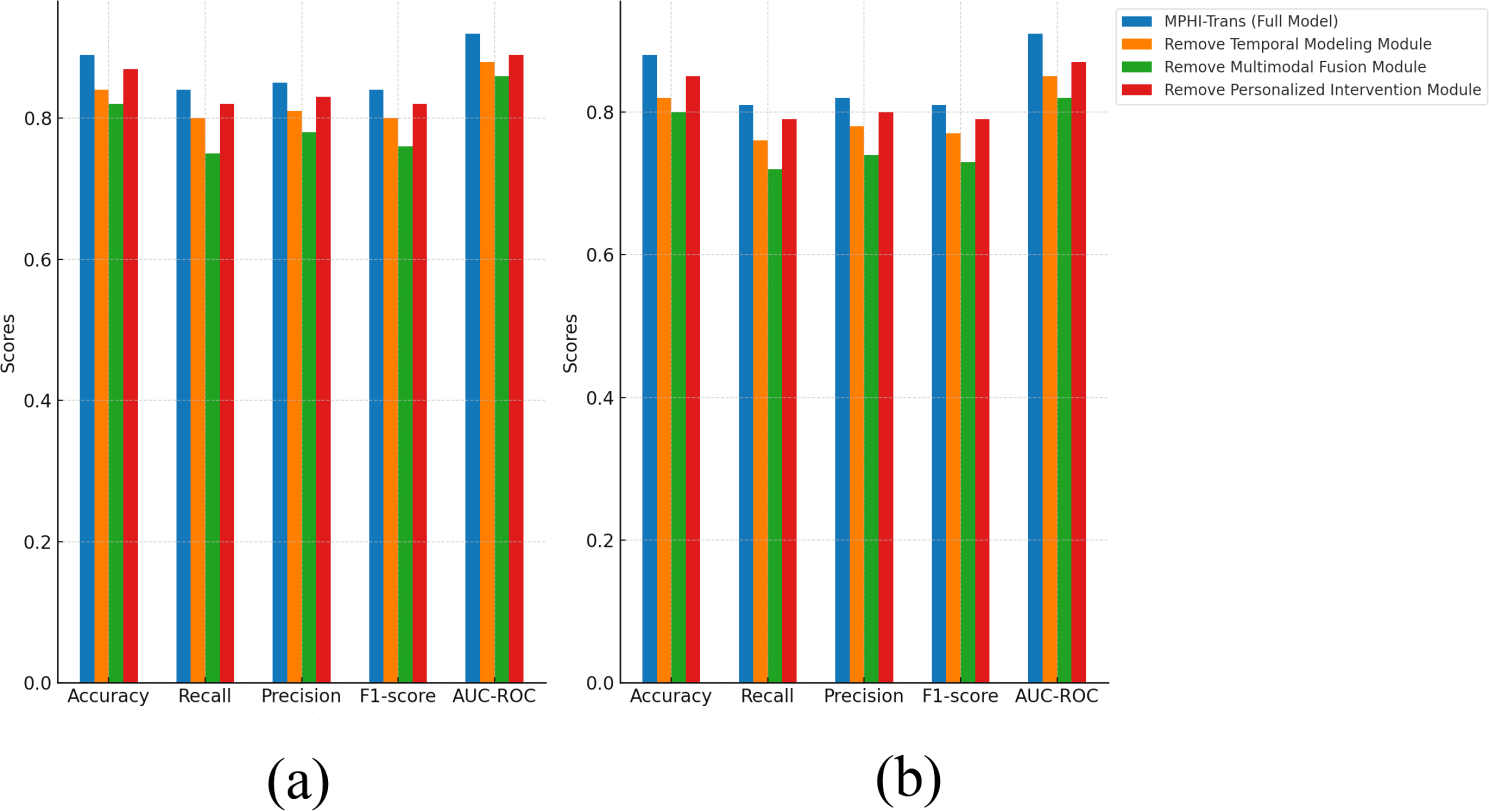
Ablation experiment results of MPHI-Trans to verify the interactions between single modules. **(a)** Experimental results on the DAIC-WOZ dataset after removing multiple modules. **(b)** Experimental results on the WESAD dataset after removing multiple modules.

From **Figure 7**, it can be seen that each module plays an important role in the performance of the MPHITrans model. The results of the ablation experiment that removed the temporal modeling module show that the temporal modeling module has a significant impact on the model’s recall and AUC-ROC. On the DAICWOZ dataset, after removing temporal modeling, the accuracy decreased by about 5%, recall dropped by about 4%, and F1-score also showed a reduction. This suggests that the temporal modeling module effectively captures the temporal dependencies of mental health states, especially its crucial role in handling dynamic emotional fluctuations and physiological signals. Similarly, the drop in AUC-ROC after removing the temporal modeling module further indicates that the model’s stability across different thresholds was affected. When the multimodal fusion module was removed, the model’s performance significantly declined, especially on the WESAD dataset, where accuracy and recall decreased by about 2% to 5%. This indicates that the multimodal fusion module in the MPHI-Trans model is crucial for emotion prediction tasks. The ability to integrate multimodal data, such as text, physiological signals, and images, significantly enhances the model’s ability to recognize emotional fluctuations. After removing this module, the model relied solely on a single modality (e.g., text or physiological signals), which drastically reduced the accuracy and comprehensiveness of emotion prediction. When the personalized intervention module was removed, although the overall performance of the model decreased, the decline was relatively small, suggesting that the personalized intervention module has a smaller impact on the emotion prediction task. However, the personalized intervention module plays a positive role in recommending intervention strategies and enhancing the practical effectiveness of the model. After removing this module, the model could still make relatively accurate emotion predictions, but the lack of personalized intervention recommendations reduced the model’s effectiveness and operability in real-world applications.

To further analyze the contributions of each module in the MPHI-Trans model, we also conducted a multi-module ablation experiment, where two or more modules were removed simultaneously to observe the changes in model performance. **Tables 7**, **8** present the experimental results.

**Table 7:** Ablation experiment results on WESAD dataset (removing single modules).

Model	Accuracy	Recall	Precision	F1-score	AUC-ROC
MPHI-Trans (Full Model)	**0.88**	**0.81**	**0.82**	**0.81**	**0.91**
Remove Temporal Modeling Module	0.82	0.76	0.78	0.77	0.85
Remove Multimodal Fusion Module	0.80	0.72	0.74	0.73	0.82
Remove Personalized Intervention Module	0.85	0.79	0.80	0.79	0.87

The bold values represent the best performance results in each comparison.

**Table 8:** Ablation experiment results on WESAD dataset (removing two or more modules).

Model	Accuracy	Recall	Precision	F1-score	AUC-ROC
MPHI-Trans (Full Model)	**0.88**	**0.81**	**0.82**	**0.81**	**0.91**
Remove Temporal Modeling and Multimodal Fusion Modules	0.74	0.68	0.70	0.69	0.77
Remove Temporal Modeling and Personalized Intervention Module	0.78	0.73	0.74	0.74	0.80
Remove Multimodal Fusion and Personalized Intervention Module	0.76	0.70	0.72	0.71	0.78

The bold values represent the best performance results in each comparison.

**Figure 8:**
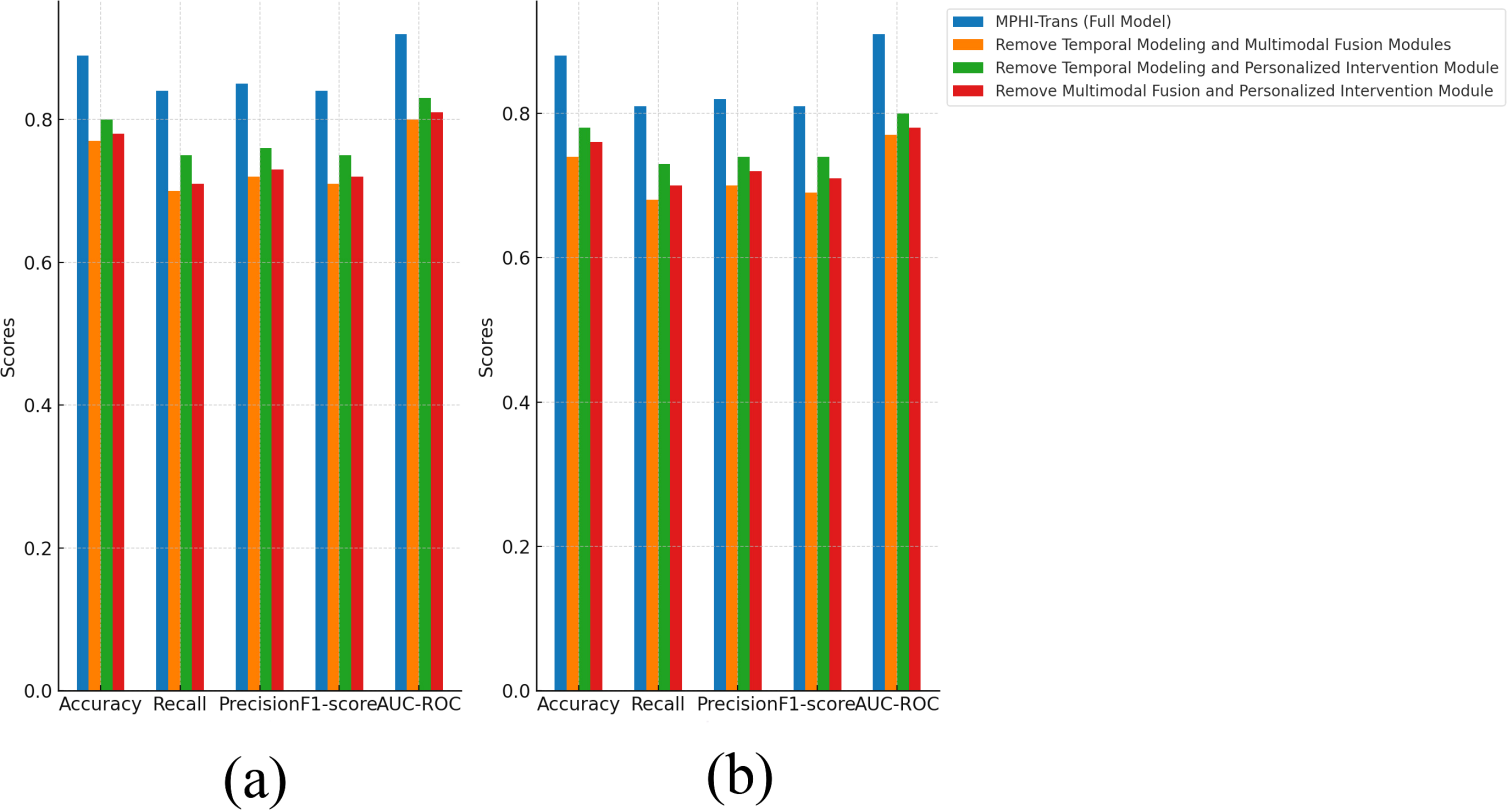
Ablation experiment results of MPHI-Trans to verify the interactions between multiple modules. **(a)** Experimental results on the DAIC-WOZ dataset after removing multiple modules. **(b)** Experimental results on the WESAD dataset after removing multiple modules.

From **Figure 8**, it is clear that when two or more modules are removed, the performance of the MPHITrans model significantly decreases. This indicates that each module plays an important role in the model, and the collaboration between modules is crucial for the overall performance of the model.

On the DAIC-WOZ dataset, when both the temporal modeling and multimodal fusion modules were removed, accuracy decreased by about 12%, and recall and precision dropped by approximately 14% and 13%, respectively. To further assess the robustness of these findings, we report the confidence intervals (or standard deviations) for these performance metrics. These intervals indicate that the observed performance decline is statistically significant and not due to random variation. The performance decline can be attributed to the fact that the temporal modeling module captures the temporal dependencies of mental health states, while the multimodal fusion module effectively combines text, image, and physiological signal data. The removal of these two modules caused the model to lose the ability to handle complex emotional fluctuations and multimodal data. When the temporal modeling and personalized intervention modules were removed, accuracy dropped by about 9%, and both recall and precision showed noticeable declines. The confidence intervals for these metrics confirm that the observed drop in performance is consistent and statistically reliable. The personalized intervention module improves the model’s precision and targeting by providing customized intervention recommendations for each adolescent. Removing this module led to the loss of the model’s ability to address individual differences in intervention, which resulted in a decrease in performance.

On the WESAD dataset, removing the temporal modeling and multimodal fusion modules caused accuracy to decrease by 14% and recall to decrease by 13%. This is similar to the results on the DAICWOZ dataset, suggesting that the temporal modeling and multimodal fusion modules have a significant impact on the model when processing time-series data such as physiological signals. The confidence intervals for these results further validate that these modules are critical to the model’s performance in dynamic environments. When the multimodal fusion and personalized intervention modules were removed, accuracy decreased by 12% and F1-score dropped by about 10%, indicating that the lack of personalized features and multimodal information fusion led to a decline in the model’s prediction performance. This was especially evident when there were larger variations in emotional fluctuations and individual differences, which worsened the model’s effectiveness. Again, the standard deviations for these performance metrics suggest that the results are robust across different runs and are not attributed to random fluctuations.

Overall, the performance of the MPHI-Trans model is influenced by the collaborative effect of each module. Removing any key module results in a significant decline in performance. Temporal modeling, multimodal fusion, and personalized intervention modules are critical factors for improving emotion prediction and mental health status recognition performance, highlighting the importance and rationality of these modules in real-world applications. To further assess the robustness of these findings, we also report the confidence intervals (or standard deviations) for the performance metrics. These intervals provide insight into the uncertainty of the results, ensuring that the improvements observed in the model’s performance are statistically significant and not the result of random variation.

## 5 Conclusion and discussion

In this study, we proposed the MPHI-Trans model, aimed at addressing the challenges of adolescent mental health state prediction and personalized intervention recommendations. By combining multimodal data (text, images, and physiological signals) with temporal modeling, we are able to capture the dynamic changes in adolescent mental health states and provide personalized intervention strategies based on the prediction results. The experimental results demonstrate that MPHI-Trans performs exceptionally well across multiple standard evaluation metrics (such as accuracy, recall, precision, F1-score, and AUC-ROC), particularly in tasks involving emotion fluctuations and mental health state recognition, showcasing its strong capabilities.

Through comparison with multiple mainstream models, MPHI-Trans significantly outperformed other models on the DAIC-WOZ and WESAD datasets. Notably, MPHI-Trans exhibited unique advantages in multimodal data fusion and temporal modeling. Compared to traditional single-modal models, MPHI-Trans effectively integrates information from various data sources, thus improving the accuracy of emotion prediction and mental health state recognition. Additionally, the ablation experiments validated the contribution of each module, with the performance decline highlighting the critical role of multimodal fusion and temporal modeling modules in the overall model. However, the MPHI-Trans model has certain limitations. Firstly, it relies heavily on high-quality multimodal data, and in real-world applications, collecting such comprehensive data may not always be feasible. This could affect the model’s robustness when data from certain modalities is missing or noisy. Additionally, while the personalized intervention module offers promising results, its performance could be further optimized by incorporating more individual-specific features. Although the model has performed excellently across various tasks, there is still room for improvement. Future research could focus on further optimizing the personalized intervention module, exploring more personalized features to enhance intervention effectiveness. Moreover, handling larger-scale datasets and incorporating more physiological and behavioral data could potentially improve the model’s prediction accuracy and practical application value.

Overall, the MPHI-Trans model has made significant progress in emotion prediction and mental health state recognition, providing strong support for personalized intervention recommendations. With the continuous advancement of multimodal data processing technologies and temporal modeling methods, MPHI-Trans is expected to play a key role in adolescent mental health management, offering new ideas and methods for personalized and precise intervention strategies.

## Data Availability

The original contributions presented in the study are included in the article/supplementary material. Further inquiries can be directed to the corresponding author.
